# Transcriptomics of Diphyllatea (CRuMs) from South Pacific crater lakes confirm new cryptic clades

**DOI:** 10.1111/jeu.13060

**Published:** 2024-09-28

**Authors:** Luis Javier Galindo, Varsha Mathur, Hadleigh Frost, Guifré Torruella, Thomas A. Richards, Nicholas A. T. Irwin

**Affiliations:** ^1^ Department of Biology University of Oxford Oxford UK; ^2^ Instituto Universitario de Investigación del Agua Universidad de Granada Granada Spain; ^3^ Departamento de Ecología, Facultad de Ciencias Universidad de Granada Granada Spain; ^4^ Merton College University of Oxford Oxford UK; ^5^ Institut de Biologia Evolutiva (CSIC‐UPF) Barcelona Catalonia Spain; ^6^ Gregor Mendel Institute (GMI) Austrian Academy of Sciences, Vienna BioCenter (VBC) Vienna Austria

**Keywords:** biogeography, *Collodictyon*, crater lakes, Diphyllatea, phylogenomics, single‐cell, transcriptomics

## Abstract

The Diphyllatea (CRuMs) are heterotrophic protists currently divided into three distinct clades (Diphy I–III). Diphy I are biflagellates in the genus *Diphylleia*, whereas Diphy II and III represent cryptic clades comprising *Collodictyon*‐type quadriflagellates that were recently distinguished based on rRNA gene phylogenies. Here, we isolated Diphyllatea from freshwater crater lakes on two South Pacific islands and generated high‐quality transcriptomes from species representing each clade, including the first transcriptomic data from Diphy III. Phylogenomic analyses support the separation of Diphy II and III, while transcriptome completeness highlights the utility of these data for future studies. Lastly, we discuss the biogeography and ecology of Diphyllatea on these remote islands.

## INTRODUCTION

CRuMs is a eukaryotic supergroup composed of morphologically diverse heterotrophic organisms (Brown et al., 2018; Zhao et al., 2012). The CRuMs branch sister to the Amorphea (Brown et al., 2018; Burki et al., 2020), making them an important clade for reconstructing early eukaryotic evolution (Blaz et al., 2023). The diversity of cellular forms within CRuMs includes filopodial amoebas within the Rigifilida (Mikrjukov & Mylnikov, 2001; Yabuki et al., 2013), gliding nanoflagellates within the Mantamonadida (Blaz et al., 2023; Cavalier‐Smith et al., 2014; Glücksman et al., 2011), and large multiflagellated predatory swimming protists within the class Diphyllatea (Brugerolle, 2006; Brugerolle et al., 2002; Orr et al., 2018). Based on morphological observations and SSU (small subunit) rRNA gene phylogenies, the class Diphyllatea (order Diphylleida) has been proposed to group the families Diphylleidae and Sulcomonadidae. The Diphylleidae includes the biflagellate, *Diphylleia rotans*, and the quadriflagellate, *Collodictyon triciliatum* (Cavalier‐smith, 2013; Ruggiero et al., 2015; Zhao et al., 2012), whereas the Sulcomonadidae is represented by the biflagellate species, *Sulcomonas lacustris* (Brugerolle, 2006).

A recent advance in the taxonomy of Diphyllatea came following the isolation and morphological characterization of 11 new isolates (Orr et al., 2018). Orr et al. (2018) sequenced the SSU rRNA genes of these organisms and generated the most complete phylogeny, in terms of diversity, of the group to date. Based on this phylogeny, the authors proposed that Diphyllatea is composed of three major clades: Diphy I, II, and III. Diphy I corresponds to the biflagellated *Diphylleia*, while clades II and III are sister groups comprising cryptic quadriflagellated species with a *Collodictyon triciliatum*‐type morphology. Importantly, this work identified Diphy III as a distinct clade at the same taxonomic level as Diphy I and II. Given SSU rRNA gene sequence divergence between Diphy II and III (79–89% sequence identity), the authors argued that these two cryptic clades, which were morphologically indistinguishable using light microscopy, could represent different genera (Orr et al., 2018). However, the SSU rRNA gene can misrepresent microbial species diversity (Piganeau et al., 2011), and there is little molecular data available from the Diphyllatea for confirmatory phylogenomic analyses or wider comparative genomic studies. Currently, there is only one high‐quality transcriptome available for *D. rotans* (Brown et al., 2018) (Diphy I clade) and a few 454 pyrosequencing contigs from *C. triciliatum* (Diphy II clade) (Zhao et al., 2012). Accordingly, we lack the data required to confirm the phylogenetic relationships within the Diphyllatea and understand CRuMs evolution more broadly.

To address this, we generated four new transcriptomes representing each of the three clades of Diphyllatea, with the aim of reconstructing the evolutionary relationships within the group and improving the availability of CRuMs transcriptomic data. To accomplish this, we isolated single cells from water samples derived from four freshwater volcanic crater lakes on two geographically distant South Pacific islands, Uvea (Wallis & Futuna) and Upolu (Samoa). These transcriptomes include one representative of Diphy I, two from Diphy II, and the first transcriptome from Diphy III. These high‐quality transcriptomes will provide a useful resource for future genomic studies and permitted phylogenomic analyses, confirming that Diphy III is a distinct clade sister to Diphy II. Lastly, we hypothesize about how these freshwater protists colonized the remote and isolated crater lakes in the South Pacific and discuss their potential ecological role as microbial predators and grazers.

## MATERIALS AND METHODS

### Sample collection, cell isolation, transcriptome sequencing, and assembly

**FIGURE 1:**
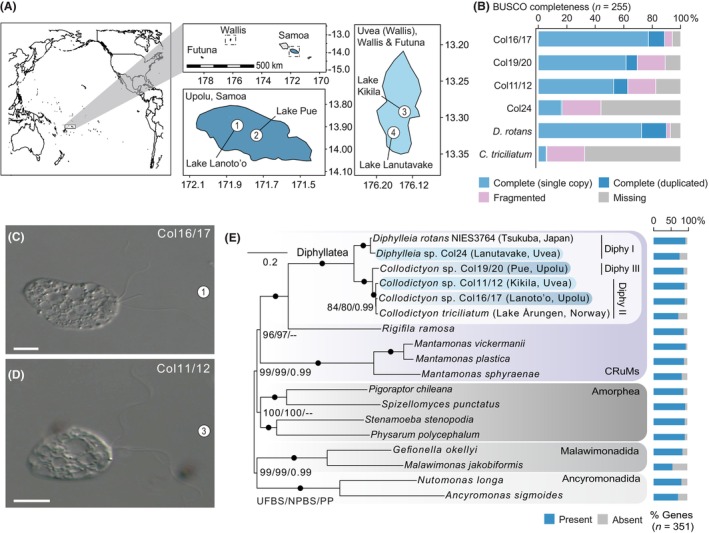
(A) Maps depicting the locations of the sampled lakes on the islands of Upolu (Samoa) and Uvea (Wallis & Futuna). Numbers correspond to each of the lakes: Lake Lanoto'o (1), Lake Pue (2), Lake Kikila (3) and Lake Lanutavake (4). (B) BUSCO completeness bar charts for all the transcriptomic data available for the Diphyllatea based on the eukaryotic_odb10 dataset. (C, D) DIC light micrographs of *Collodictyon* sp. cells (Diphy II) from Lakes Lanoto'o (C) and Kikila (D); scale bars 10 μm. (E) Maximum‐likelihood phylogenomic tree of CRuMs and their relatives constructed using a dataset comprising 126,700 amino acid positions derived from 351 concatenated proteins, representing 18 CRuMs species and their relatives. The phylogeny was inferred using the PMSF approximation of the LG + C60 + G model and statistical support was assessed using ultrafast bootstraps (UFBS) and nonparametric bootstraps (NPBS). Bayesian posterior probabilities (PP) are also shown and were inferred using the CAT‐Poisson model. Nodes with full statistical support are indicated with black circles and the scale bar represents the average number of substitutions per site. Light and dark blue coloring corresponds to the island of Uvea (Wallis & Futuna) and Upolu (Samoa), respectively.

Water samples were collected from freshwater volcanic lakes on the islands of Uvea (Wallis and Futuna) and Upolu (Samoa) between the 24th of April and 15th of May of 2023 as part of an effort to characterize the protist diversity of volcanic crater lakes in the South Pacific. The water samples used in this study were collected from lakes Kikila (surface water, first 10 cm; 13°17′48.3″ S 176°11′24.7″ W) and Lanutavake (3 m depth; 13°19′16.2″ S 176°12′50.6″ W) on the island of Uvea (Wallis and Futuna) and from lakes Lanoto'o (at 1.5 m depth; 13°54′39.6″ S 171°49′40.1″ W) and Pue (at 3 m depth; 13°56′13.0″ S 171°45′14.7″ W) on the island of Upolu (Samoa) (Figure 1A). Water was collected using a 5 L Niskin water sampler deployed from an inflatable kayak. For each sample, 40 mL of lake water was stored in a 25 cm^2^ vented cell culture flask at room temperature and taken to the laboratory for further analysis.

Diphyllatea cells were identified based on morphology using an Olympus CKX53 inverted microscope, and single cells were isolated using glass capillary micropipettes. We generated two samples per lake, which each contained pools of 3–7 morphologically consistent cells for downstream cDNA synthesis using the Smart‐Seq2 protocol (Picelli et al., 2014). The cells were washed at least three times in sterile molecular‐grade water (Fisher Scientific), photographed when possible, and transferred to a 0.2 mL thin‐walled PCR tube containing 2 μL of cell lysis buffer (0.2% Triton X‐100 and RNase inhibitor (Invitrogen)). The samples were incubated with free dNTPs and the oligo‐dT primer (5′–AAGCAGTGGTATCAACGCAGAGTACT_30_VN‐3′), which anneals to RNA molecules containing a poly(A) tail. Next, the reverse transcription (RT) reaction was performed using Superscript II reverse transcriptase (Invitrogen) and a template‐switching oligo (TSO) (5′‐AAGCAGTGGTATCAACGCAGAGTACATrGrG+G‐3′). The resulting cDNA was amplified using the IS PCR primer (5′ AAGCAGTGGTATCAACGCAGAGT‐3′) and KAPA HiFi HotStart ReadyMix (2X; KAPA Biosystems) for 18 PCR cycles. All steps were performed using reaction mixes and thermal cycler conditions described in the Smart‐Seq2 protocol (Picelli et al., 2014). Finally, the cDNA was purified using Ampure XP beads (Beckman Coulter), and the cDNA concentration was quantified with a Qubit 2.0 fluorometer (Thermo Fisher Scientific Inc.). Prior to high‐throughput sequencing, 2 μL of the final cDNA product was used as a template for PCR amplification of the 18S rRNA gene. GoTaq DNA Polymerase (Thermo Fisher Scientific) and the general eukaryotic primers 82F (5′‐GAAACTGCGAATGGCTC‐3′) and 1520R (5′‐CYGCAGGTTCACCTAC‐3′) were used with an annealing temperature of 55°C (30 s) and an extension time of 2 min (72°C). PCR products were sequenced using Sanger sequencing (performed by Eurofins), and sequences were identified by performing BLASTn searches against the nonredundant NCBI database (Pruitt et al., 2007). After identification, sequencing libraries were prepared using the Novogene NGS RNA library prep set protocol (PT042) and sequenced on a single lane of an Illumina Novoseq 6000 using 150‐bp paired‐end reads. All raw reads have been deposited in NCBI Bioproject PRJNA1100983.

The quality of the raw sequencing reads was assessed using FastQC v0.12.1 (Andrews, 2010). Low‐quality reads, adapters, and primer sequences were trimmed using Trimmomatic v0.39, and the remaining reads were assembled with rnaSpades v3.13.0 (Bolger et al., 2014; Bushmanova et al., 2019). To remove bacterial contamination, both BLASTn searches against the NCBI nt database and Diamond v2.1.7 BLASTx searches against UniProt reference proteomes were conducted (Buchfink et al., 2021; Consortium, 2022). The bacterial reads were filtered out using BlobToolKit v4.2.1 and Bowtie v2.5.1 (Laetsch & Blaxter, 2017; Langmead et al., 2009). Given our SSU rRNA gene sequencing results, we combined assembled transcripts derived from similar strains (SSU rRNA gene sequence similarity >99%) that were obtained from the same lake sample. Accordingly, we generated the following four merged Diphyllatea transcriptomes: Col11/12 (Lake Kikila), Col16/17 (Lake Lanoto'o), Col19/20 (Lake Pue), and Col24 (Lake Lanutavake). Protein coding regions were predicted using TransDecoder v5.7.1 (https://github.com/TransDecoder/TransDecoder) and clustered with CD‐HIT v4.8.1 (Li & Godzik, 2006) at 99% identity. To maximize sensitivity for capturing ORFs, Diamond v2.1.7 BLASTx searches against the UniProt database were carried out to retain ORFs with homology to known proteins (Buchfink et al., 2021; Consortium, 2022). The completeness of the predicted proteomes was assessed using BUSCO v5.5.0 (Manni et al., 2021; Simão et al., 2015) using the eukaryota_odb10 lineage dataset. Assembled transcripts and proteomes are available in a Figshare data repository (https://figshare.com/projects/Diphyllatea_from_the_South_Pacific/200110).

### SSU rRNA gene phylogeny and phylogenomic analyses

To identify the species isolated for transcriptomics, the Diphyllatea SSU rRNA gene dataset developed by Orr et al. (2018) was expanded with SSU sequences extracted from the sequenced transcriptomes. These 38 sequences were aligned with MAFFT v.7 (Katoh & Standley, 2013) and trimmed with trimAL v.1.2 (Capella‐Gutiérrez et al., 2009) using a gap‐threshold of 30%, resulting in an alignment with 1078 sites. The resulting alignment was used to generate a maximum likelihood phylogeny with IQ‐TREE v.1.6.11 (Nguyen et al., 2015) using the GTR + F + I + R2 model, selected using ModelFinder (Kalyaanamoorthy et al., 2017) based on Bayesian information criteria (BIC). Statistical support was inferred from 1000 ultra‐fast bootstraps.

Using the transcriptomic data, we also performed a multigene phylogenomic analysis using a CRuMs‐enriched dataset comprising 351 conserved protein markers from 18 species (Blaz et al., 2023; Lax et al., 2018). Each protein marker was aligned with MAFFT v.7 and trimmed with trimAL v.1.2 with the automated1 option. Single‐protein trees were inferred from each trimmed alignment using FastTree v2.1.11 (Price et al., 2009). Alignments and trees were manually inspected to discard contaminants and paralogs and were edited using Geneious v11.0.18 (Kearse et al., 2012). Representative taxa were then selected (10 CRuMs +8 outgroups from Amorphea, Ancyromonadida, and Malawimonadida), and the resulting proteins were realigned and trimmed as stated above before finally being concatenated with alvert.py from the barrel‐o‐monkeys package (http://rogerlab.biochemistryandmolecularbiology.dal.ca/Software/Software.htm#Monkeybarrel). This resulted in a concatenated matrix with 126,700 amino acid positions.

The maximum likelihood (ML) phylogenomic trees were inferred using IQ‐TREE v1.6 using the PMSF approximation (Wang et al., 2018) with a guide tree using the LG + C60 + G model. Statistical support was generated from two reconstruction rounds with 1000 ultrafast bootstraps and 100 nonparametric bootstraps. All ML substitution models were selected using ModelFinder based on BIC. Bayesian inference was performed using PhyloBayes‐MPI v1.5a (Lartillot et al., 2013). Four MCMC chains were run for 10,000 generations using the CAT‐Poisson model, sampling one every tenth tree. Of the four chains, three properly converged (max difference = 0.0277) as assessed using Tracer and PhyloBayes using a burn‐in of 25% (Rambaut et al., 2018). Phylogenies were visualized using FigTree v.1.4.4 (http://tree.bio.ed.ac.uk/software/figtree/). All alignments and trees are available in a Figshare data repository (https://figshare.com/projects/Diphyllatea_from_the_South_Pacific/200110).

## RESULTS AND DISCUSSION

### Four new transcriptomes of Diphyllatea

To investigate the phylogenetic relationships amongst the Diphyllatea, we sequenced seven transcriptomes of Diphyllatea, including two from Lake Kikila (Col11 and 12), two from Lake Lanoto'o (Col16 and 17), two from Lake Pue (Col19 and 20), and one from Lake Lanutavake (Col24) (Figure 1A). Based on relatedness (SSU > 99% pairwise identity), SSU rRNA gene phylogeny (Figure S1), and sample locality, we merged the transcripts of Col11/12, Col16/17, and Col19/20, resulting in our final dataset of four transcriptomes, including Col24. After removing bacterial contamination, we retrieved high BUSCO presence values (89% to 94.2%) for three of our transcriptomic datasets, which is a significant improvement from the 22.6% BUSCO completeness score from the currently available 454 contigs of *C. triciliatum* (Blaz et al., 2023; Zhao et al., 2012) (Figure 1B; Table S1). In contrast, Col24 (our nonmerged sample) had a lower BUSCO presence of 44%. However, Col24 is closely related to *Diphylleia* (Diphy I; SSU pairwise identity of 99.7%), for which there is a good‐quality transcriptome available (Brown et al., 2018) with a BUSCO presence of over 90% (Blaz et al., 2023). Our results highlight that single‐cell transcriptome data can be improved by pooling isolated cells or data derived from individual cells, as has been observed in other studies (Keeling & Del Campo, 2017; Mangot et al., 2017). Given their quality, these new Diphyllatea transcriptomes will also provide a valuable resource for future comparative genomic studies.

### The phylogeny of Diphyllatea

Phylogenetic analysis of the SSU rRNA gene from the new Diphyllatea transcriptomes was congruent with the analyses of Orr et al. (2018) and supported the presence of three distinct Diphyllatea clades (Figure S1). Col24 groups within Diphy I, along with the genus *Diphylleia*, whereas Col11, 12, 16, and 17 group within Diphy II alongside *Collodictyon*‐type quadriflagellated species such as *C. triciliatum*. Notably, Col19 and 20 branch sister to Diphy II in the recently identified Diphy III clade. Although we could only obtain light microscopy images of cells from the Col 11/12 and 16/17 samples (Diphy II; Figure 1C,D), our microscopy observations without imaging confirmed that the cells isolated from Diphy I and III were biflagellated and quadriflagellated, respectively. These results therefore corroborate the identification of Diphy III as an independent clade of cryptic quadriflagellated *Collodictyon*‐type species.

To support these results, we generated a concatenated multi‐gene matrix for phylogenomic analysis. Both maximum likelihood and Bayesian inference analysis using the phylogenomic matrix strongly supported the placement of all four new transcriptomes within the three clades of Diphyllatea, confirming the genetic distinctiveness of Diphy III and its sister relationship with Diphy II (Figure 1E). Taken together, these results support the establishment of new taxonomic assignments for Diphy II‐III, possibly as new genera as suggested by Orr et al. (2018). However, future work focusing on the isolation and morphological characterization of Diphy II‐III species, particularly at an ultrastructural level, will be required before formal taxonomic classifications can be made. Moreover, it will be essential to obtain molecular data from *Sulcomonas lacustris* (Sulcomonadidae) to confirm its relationship within the Diphyllatea.

### Diphyllatea in crater lakes of South Pacific islands

Diphyllatea are globally distributed in freshwater environments (Orr et al., 2018). However, it is interesting to find these exclusively freshwater protists in remote and isolated crater lakes in two different South Pacific islands, hundreds of kilometers away from each other and alternative terrestrial water systems. The long‐range dispersal and biogeography of microbial eukaryotes has been extensively discussed previously and is thought to be influenced by factors such as cell size, environmental tolerance, and the ability to encyst (Bamforth, 1981; Bass et al., 2007; Finlay, 2002; Foissner, 2006, 2007). The introduction of Diphyllatea to these lakes must have involved long‐distance travel and would have required overcoming significant environmental barriers, particularly given that the ancestor of Diphyllatea and Rigifilida was likely adapted to freshwater (Orr et al., 2018; Yabuki et al., 2013). However, the Diphyllatea are known to produce thick‐walled resting cysts, which could have facilitated their dispersal (Foissner, 2007; Orr et al., 2018). The remoteness of these crater lakes complicates human‐mediated dispersal, suggesting that their colonization could have been via natural mechanisms such as wind or animal vectors like marine birds. This dispersal ability, in combination with the known role of Diphyllatea as predators and grazers of (mainly) algae and cyanobacteria (e.g., Klaveness, 1995; Orr et al., 2018; Zhao et al., 2012), suggests that these protists could serve an important role in the establishment of trophic networks in emerging freshwater ecosystems. Lastly, our observations confirm that Diphyllatea represent a useful study group for tracking how freshwater protists can disperse across wide biogeographical territories and significant environmental boundaries.

## Supporting information


Figure S1

Table S1

## Data Availability

Additional supporting information can be found online in the Figshare repository (https://figshare.com/projects/Diphyllatea_from_the_South_Pacific/200110) and in Table S1 and Figure S1 at the end of this article. All raw reads have been deposited in NBCI under Bioproject PRJNA1100983 and the SSU rRNA gene sequences under accession numbers: PP806894, PP806898, PP809654, PP809657, PP811629, PP812520, and PP812521.
