# Supplementary material for: Transcriptomics of Diphyllatea (CRuMs) from South Pacific crater lakes confirm new cryptic clades
Source: J Eukaryot Microbiol. 2024 Sep 28;71(6):e13060. doi: 10.1111/jeu.13060 (PMC11603278; doi:10.1111/jeu.13060)
Supplement: Supplementary file 1 — Figure S1 [file JEU-71-e13060-s001.pdf]

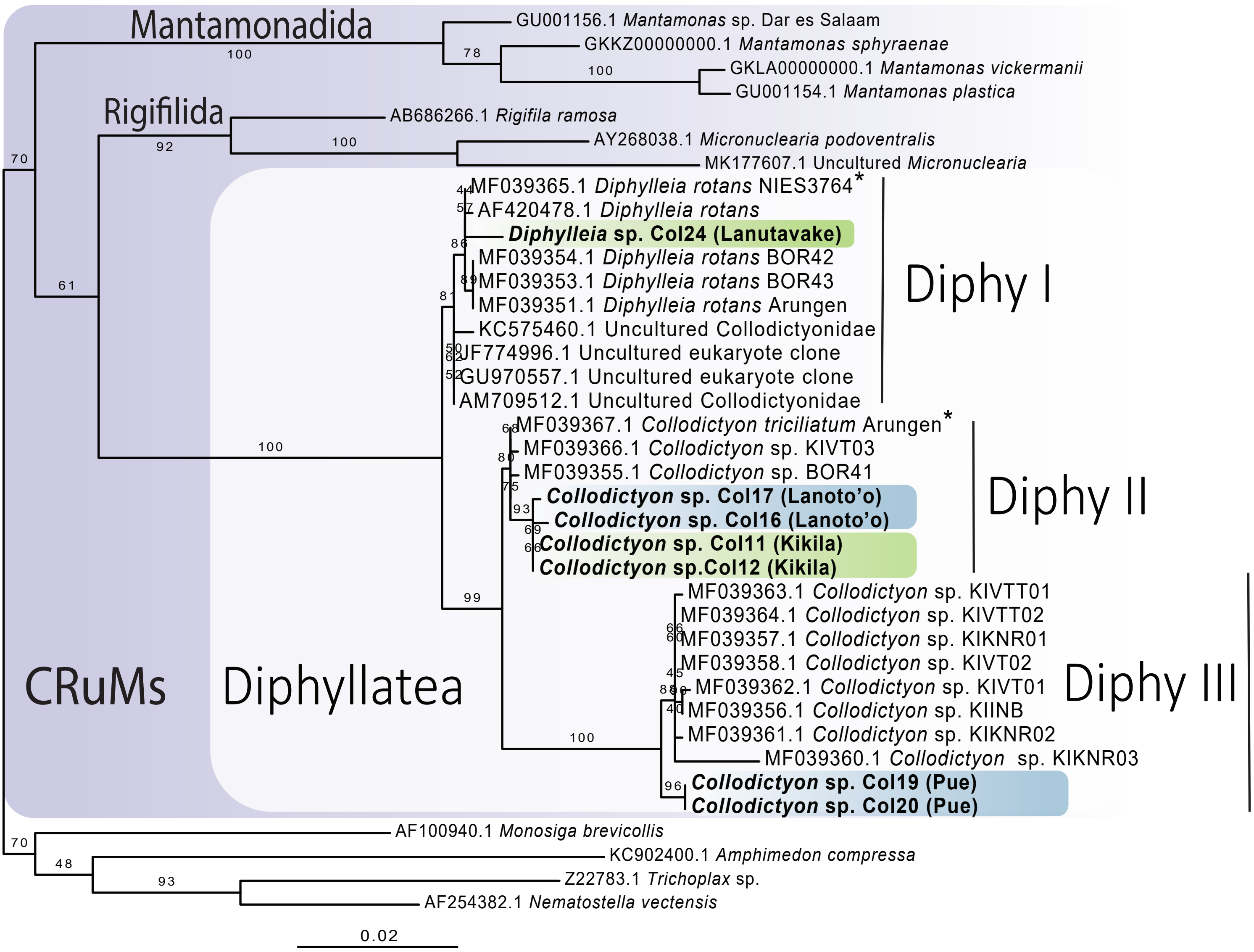

Supplementary Figure 1. 18S rRNA gene maximum likelihood (ML) tree of the Diphyllatea including sequences for our 7 single-cell transcriptomes of Diphyllatea. The alignment includes 38 sequences with a total of length of 1,078 sites. Green indicates samples from Wallis & Futuna and blue highlights samples from Samoa. Asterisks indicate species for which there is genomic/transcriptomic data available. The ML tree was reconstructed under the GTR+F+I+R2 model.
