# Supplementary material for: Transcriptomics of Diphyllatea (CRuMs) from South Pacific crater lakes confirm new cryptic clades
Source: J Eukaryot Microbiol. 2024 Sep 28;71(6):e13060. doi: 10.1111/jeu.13060 (PMC11603278; doi:10.1111/jeu.13060)
Supplement: Supplementary file 2 — Table S1 [file JEU-71-e13060-s002.pdf]

Supplementary Table 1. Sequencing and assembly information of the Diphyllatea transcriptomes sequenced in this study

Sample Name	Species	Sample ID	RNA Kit	Library Prep Kit	Illumina Sequencer
Col_Kiki_11	Collodyction sp.	Col11	Smart-Seq 2	Novogene NGS RNA Library Prep Set (PT042)	Novaseq 6000
Col_Kiki_12	Collodyction sp.	Col12	Smart-Seq 2	Novogene NGS RNA Library Prep Set (PT042)	Novaseq 6000
Col_Lano_16	Collodyction sp.	Col16	Smart-Seq 2	Novogene NGS RNA Library Prep Set (PT042)	Novaseq 6000
Col_Lano_17	Collodyction sp.	Col17	Smart-Seq 2	Novogene NGS RNA Library Prep Set (PT042)	Novaseq 6000
Col_Pue_19	Collodyction sp.	Col19	Smart-Seq 2	Novogene NGS RNA Library Prep Set (PT042)	Novaseq 6000
Col_Pue_20	Collodyction sp.	Col20	Smart-Seq 2	Novogene NGS RNA Library Prep Set (PT042)	Novaseq 6000
Dip_Lanu_24	Diphylleia sp.	Col24	Smart-Seq 2	Novogene NGS RNA Library Prep Set (PT042)	Novaseq 6000

Trimmomatic (adapter and PCR primer removal)

Sample ID	Input Read Pairs	Both Surviving	Forward Only Surviving	Reverse Only Surviving	Dropped
Col11	14183303	3104008 (21.88%)	5487049 (38.69%)	5358396 (37.78%)	233850 (1.65%)
Col12	14451241	3307996 (22.89%)	5610637 (38.82%)	5412412 (37.45%)	120196 (0.83%)
Col16	13154779	5180774 (39.38%)	4089996 (31.09%)	3694571 (28.09%)	189438 (1.44%)
Col17	15361266	6565067 (42.74%)	4561563 (29.70%)	3892087 (25.34%)	342549 (2.23%)
Col19	11291676	3698728 (32.76%)	3079435 (27.27%)	2864802 (25.37%)	1648711 (14.60%)
Col20	13174399	4393572 (33.35%)	4012461 (30.46%)	3662371 (27.80%)	1105995 (8.40%)
Col24	13043578	4036110 (30.94%)	4466187 (34.24%)	4211468 (32.29%)	329813 (2.53%)

rnaSpades (initial denovo assembly)

Sample ID	Number of transcripts	BUSCO %	BUSCO (lineage = eukaryotes)
Col11	45690	82.8	C:60.8%[S:55.3%,D:5.5%],F:22.0%,M:17.2%,n:255
Col12	23294	81.5	C:63.5%[S:54.9%,D:8.6%],F:18.0%,M:18.5%,n:255
Col16	34058	92.9	C:83.9%[S:75.3%,D:8.6%],F:9.0%,M:7.1%,n:255
Col17	29887	95.3	C:87.5%[S:77.3%,D:10.2%],F:7.8%,M:4.7%,n:255
Col19	26009	73	C:56.9%[S:49.8%,D:7.1%],F:16.1%,M:27.0%,n:255
Col20	28089	74.5	C:58.8%[S:48.6%,D:10.2%],F:15.7%,M:25.5%,n:255
Col24	61302	52.5	C:24.3%[S:20.8%,D:3.5%],F:27.8%,M:47.9%,n:255

Combined datasets (cleaned for bacteria)

Samples	Number of transcripts	BUSCO %	
Col 11 & 12	49161	89.1	C:72.2%[S:25.1%,D:47.1%],F:16.9%,M:10.9%,n:255
Col 16 & 17	59624	95	C:89.1%[S:12.2%,D:76.9%],F:5.9%,M:5.0%,n:255
Col 19 & 20	51877	82.7	C:69.4%[S:26.7%,D:42.7%],F:13.3%,M:17.3%,n:255
Col24	26410	48.2	C:20.4%[S:18.4%,D:2.0%],F:27.8%,M:51.8%,n:255

Transdecoder: Protein prediction

Samples	Number of proteins	BUSCO %	
Col 11 & 12	31248	88.6	C:71.0%[S:27.5%,D:43.5%],F:17.6%,M:11.4%,n:255
Col 16 & 17	42837	94.1	C:89.4%[S:15.3%,D:74.1%],F:4.7%,M:5.9%,n:255
Col 19 & 20	37255	82	C:64.7%[S:25.9%,D:38.8%],F:17.3%,M:18.0%,n:255
Col 24	9864	43.5	C:19.2%[S:18.0%,D:1.2%],F:24.3%,M:56.5%,n:255

Novogene Sequencing Reads	GC (%)
FKDN230420189-1A_HY7MGDSX5_L1	41.71
FKDN230420190-1A_HY7MGDSX5_L1	43.17
FKDN230420191-1A_HY7MGDSX5_L1	42.09
FKDN230420192-1A_HGWNJDSX7_L3	46.73
FKDN230420193-1A_HFTWFDSX7_L3	42.19
FKDN230420194-1A_HFTWFDSX7_L3	40.31
FKDN230420195-1A_HY7MGDSX5_L1	39.55
